# Molecular Cloning and Expression of EG95 Gene of Iranian Isolates of *Echinococcus granulosus*

**Published:** 2012

**Authors:** S Sarvi, A Dalimi, F Ghafarifar

**Affiliations:** Department of Parasitology and Entomology, Faculty of Medical Sciences, Tarbiat Modares University, Tehran, Iran

**Keywords:** *Echinococcus granulosus*, EG95, pcDNA3 plasmid

## Abstract

**Background:**

Echinococcosis or hydatidosis is a chronic, zoonotic worldwide infection that occurs by the larval stages of taeniid cestodes of the genus *Echinococcus*. Iran is known as endemic region for this infection in the world. Vaccination has been considered as a good prevention method for this disease. Recombinant vaccines containing EG95 protein, against *E. granulosus*, has shown a high degree of protection against *E. granulosus* infection. In this study EG95 gene was extracted from Iranian isolates of *E. granulosus* and then cloned and expressed in expression vector.

**Methods:**

Protoscoleces were collected from sheep hydatid cysts. Then DNA and RNA were extracted from protoscoleces, and amplified by PCR and RT-PCR with specific primer. Afterward the purified RT-PCR products were successfully ligated into pTZ57R/T plasmid vector. The pcDNA3 plasmid was used as expression vector and Eg95 fragment sub cloned into this plasmid. The pcEG95 plasmid was digested by restriction enzymes to confirm cloning of this gene in pcDNA3 plasmid. In last step, the subcloned gene was expressed in CHO as eukaryotic cell.

**Results:**

EG95 fragment successfully was subcloned in pcDNA3 and EG95 protein was expressed by eukaryotic cell. The recombinant EG95 protein was confirmed by SDS-PAGE and Western blot.

**Conclusion:**

Recombinant plasmid of pcEG95 was constructed successfully and express of recombinant EG95 protein was confirmed.

## Introduction

Echinococcosis or hydatidosis is a chronic and zoonotic worldwide infection that occurs because of infection by the larval stages of taeniid cestodes of the genus *Echinococcus* (1, 2). *Echinococcus granulosus* is the most important cestodes in human infection. It is assumed that 50 million people are at risk of acquiring the disease in Asia and in Africa (2).

Iran is known as endemic region for the infection in the world (3). Many epidemiological studies have shown that the seroprevalence of human hydatidosis in different regions of Iran is high and between 1.2–21.4% of infectivity (4). The minimum and maximum prevalence of hydatidosis in sheep based on abattoir data are reported 5.1% (Kerman) and 74.4% (Ardabil) respectively (5, 6).

An effective vaccine against infection with *E.granulosus* would be a valuable for hydatid control campaigns (7). Early attempts at inducing immunity in dogs as definitive hosts through vaccination carry out in 1933 (8). Vaccination in sheep as intermediate host has been considered to prevention of disease in recent decades (1). In many studies, live or killed parasites were used as antigen for production of vaccine (9).

Using recombinant proteins like EG95 were assumed effective for prevention against hydatidosis. EG95 has been a highly effective sheep vaccine in New Zealand, Australia, and Argentina (10) and Iran (11) and the vaccine may prove to be a useful tool in the control of hydatid disease in areas of endemicity (9). Lightowlers et al. used this protein for vaccination of sheep against experimental challenge with the *E.granulosus*. This vaccine has shown a high degree of protection against this parasite (96–98%) (7). In other study, vaccination with EG95 obtained from three different parasite isolates conferred a high degree of protection against challenge in sheep (protection range 96–100%) (10).

Since, there was no study in Iran regarding the gene coding EG95 protein production, so we investigated to extract EG95 gene from Iranian isolates of *E. granulosus* and cloning as well as expressing it in appropriate expression vector.

## Materials and Methods

### Collection of samples

Sheep hydatid cysts were collected from a slaughterhouse in Sari City, Mazandaran Province, Iran, where the incidence of the infection is high. Protoscoleces were aspirated from cysts, pooled, and washed with sterile PBS.

### Genomic DNA extraction

DNA was extracted from protoscoleces by phenol-chloroform method (12). The DNA concentration and quality was assessed by both UV absorbance and electrophoresis on the 1% agarose gel.

### Total RNA extraction

The protoscoleces immediately after washing with PBS, was placed at the liquid nitrogen over night. Then total RNA extracted from protoscoleces with the RNX Plus kit (Cinnagene^®^) according to the manufacturer's instructions. The RNA concentration and quality was assessed by both UV absorbance and electrophoresis on the 1.5% agarose gel.

### DNA amplification

The EG95 gene of *E. granulosus* was amplified by PCR. This reaction was performed with the Bioneer master mix (AccuPower^®^ PCR PreMix, k-2012). Primers were designed according to the published *E. granulosus* EG95 vaccine antigen (EG95) cDNA sequences (GenBank accession number AY421719.1). The forward primer: CGGAATCATGGCATTCCAGTTATGTCTC, with the restriction site for *EcoRI*, and Reverse primer: GCCTCGAGTCAAGTAAGGACAAC, with the restriction site for *XhoI*. PCR procedure including initial denaturation at 94°C for 4 min, then 35 cycles of denaturation at 94°C for 1 min, annealing at 53°C for 1 min, extension 72°C for 1 min, and final extension at 72°C for 10 min. The PCR product was analyzed by electrophoresis on a 1.2% agarose gel and the size of them compared with 100 bp DNA ladder (GeneRuler™ 100 bp Plus DNA Ladder, Fermentase ^®^). This amplified DNA was sequenced with Capillary Electrophoresis System by Macrogen Company (Korea).

### RT-PCR amplification

The total RNA reverse was transcribed to cDNA (with using RevertAid™ H Minus Reverse Transcriptase, Fermentas^®^), then this cDNA was used as template DNA for RT-PCR amplification. The primers used in this reaction, was the same as the primers used in PCR reaction. RT-PCR procedure included initial denaturation at 94°C for 3 min, then 35 cycles of denaturation at 94°C for 1 min, annealing at 53°C for 40 sec, extension 72°C for 45 sec, and final extension at 72°C for 10 min. The RT-PCR product likewise the PCR product was analyzed by electrophoresis on a 1.5% agarose gel and the size of them compared with 100 bp DNA ladder, then this products were isolated and purified from agarose gel with using DNA gel Extraction Kit (AccuPrep^®^ Gel Purification Kit, Bioneer).

### Ligation and Transformation of EG95 gene

The purified RT-PCR products were ligated into pTZ57R/T plasmid vector (InsT/A cloneTM PCR product cloning Kit, Fermentas^®^) according to the manufacturer's instructions. For predispose the bacteria, we used *E. coli* Top10 strain and calcium chloride method (12). After ligation, the ligated plasmid was transformed to the competent bacteria according the protocol (12), and cultured in Luria-Bertani (LB) broth medium free antibiotic by incubation for 1h, at 37 °C. Then these bacteria were plated on the LB Agar plate containing antibiotic (ampicillin 100 mg/ml), IPTG 200 mg /ml, X-Gal 20 mg/ml and this plate was incubated at 37 °C for 16–18 h. After this time, the blue and white colony was formed at the plate. To confirm the gene cloning we used Colony-PCR method. The plasmid was extracted from bacteria by plasmid extraction kit (Accu-Prep Plasmid MiniPrep DNA Extraction Kit, Bioneer^®^), and digested by EcoRI and XhoI enzymes. The enzymatic reaction was performed in two conditions, mono and double digestion, separately. The products was analyzed by electrophoresis on a 1% agarose gel and the size of them compared with 1kb DNA ladder (GeneRuler™ 1kb, Fermentase^®^). Eg95 gene that digested from pT-Eg95 in double digest reaction, extracted from gel by using gel Extraction Kit (AccuPrep^®^ Gel Purification Kit, Bioneer^®^).

### Sub cloning of Eg95 in expression vector

The pcDNA3 plasmid was used as expression vector and Eg95 fragment sub cloned into this plasmid. The pcDNA3 plasmid was digested by the same enzymes used for digestion of pT-Eg95 (EcoR1 and XhoI). Then digested plasmids were purified from agarose gel by using DNA Extraction Kit (AccuPrep^®^ Gel Purification Kit, Bioneer). The Eg95 fragment was ligated into the digested pcDNA3 according to the same protocol that used for ligation of Eg95 and pTZ57R/T (12). The ligation product was transformed into the *E. coli* Top10 strain, according to the protocol (12) and cultured in Luria-Bertani (LB) broth medium free antibiotic by incubating for 1h at 37 °C with shaking. These transformed bacteria were plated onto LB agar plates contain ampicillin 100 mg/ml and incubating at 37 °C for 16–18h. For identifying the pcEg95-IR recombinant plasmid, we used Colony-PCR amplification and restricted digestion with the EcoRI and XhoI enzyme. The recombinant pcEg95-IR plasmids were sequenced by Macrogen Company.

### Expression of recombinant EG95 protein

pcEG95-IR was transfected into the Chinese hamster ovary cell (CHO) using Fugene 6 Transfection reagent kit (Roche). Transfected cells were seeded in a 12-well tissue culture dish with Dulbecco's Modified Eagle Medium (DMEM) contining neomycin antibiotic (G418) and then kept at 37°C in a 5% CO2 incubator.

### SDS PAGE and Western blot analysis

After 14 days, the transfected cell harvested from the media and expressed recombinant EG95 protein was examined on 15% acrylamide gel in SDS PAGE. The resulted proteins were transferred to the nitrocellulose membrane. The membranes were incubated in PBS (Phosphate Buffered Saline containing 2% BSA (Bovine Serum Albumin) and then washed three times with PBST (PBS-Tween20 0.5%.). The nitrocellulose membrane was reacted with 1:200 seropositive mouse antibody for 1 h at 37°C. Then were washed three times with PBST and subsequently treated with horseradish peroxidase (HRP) conjugated with goat anti mouse IgG with 1: 5000 dilution for 1 hour at 37°C. The page was visualized for color after development in Di Amino Benzidine/H2O2 substrate solution for 15 min at room temperature. The reaction was stopped by washing four times in distilled H2O.

## Results

**Fig. 1:**
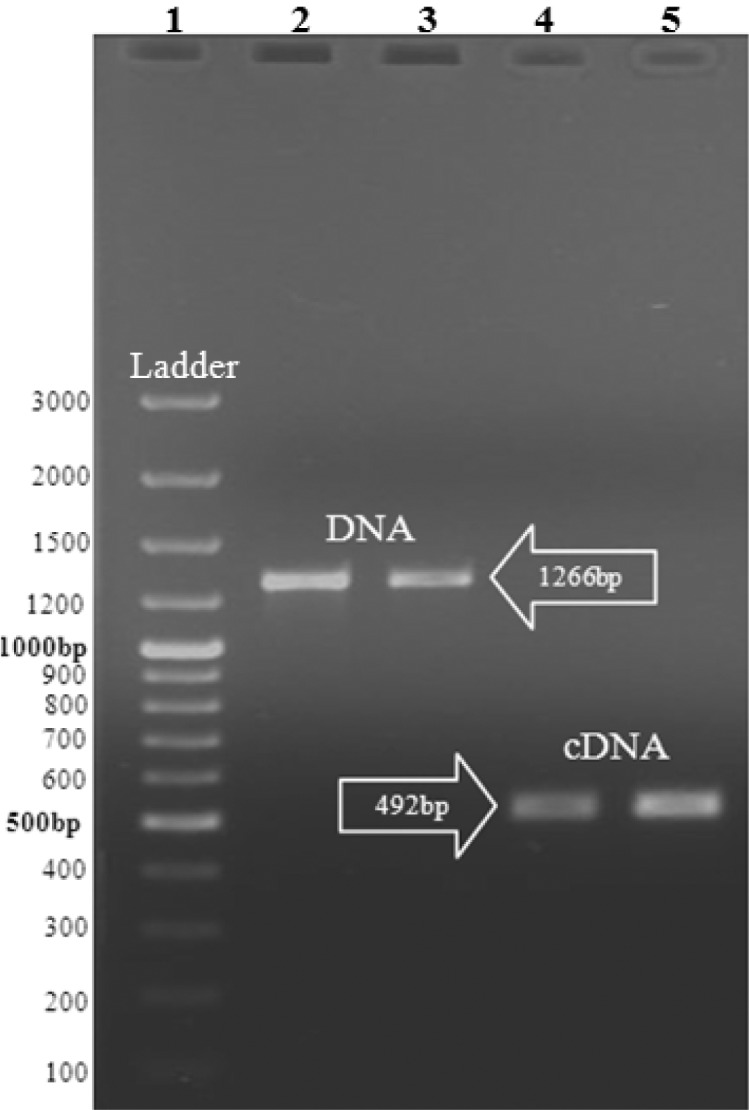
Amplification of DNA and RNA (cDNA) extracts of Iranian isolates of *E.granulosus* by RCR and RT-PCR reactions. Lane 1: Ladder, Lane 2 & 3: DNA amplification (1266 bp), Lane 4 & 5: cDNA amplification (492 bp)

**Fig. 2:**
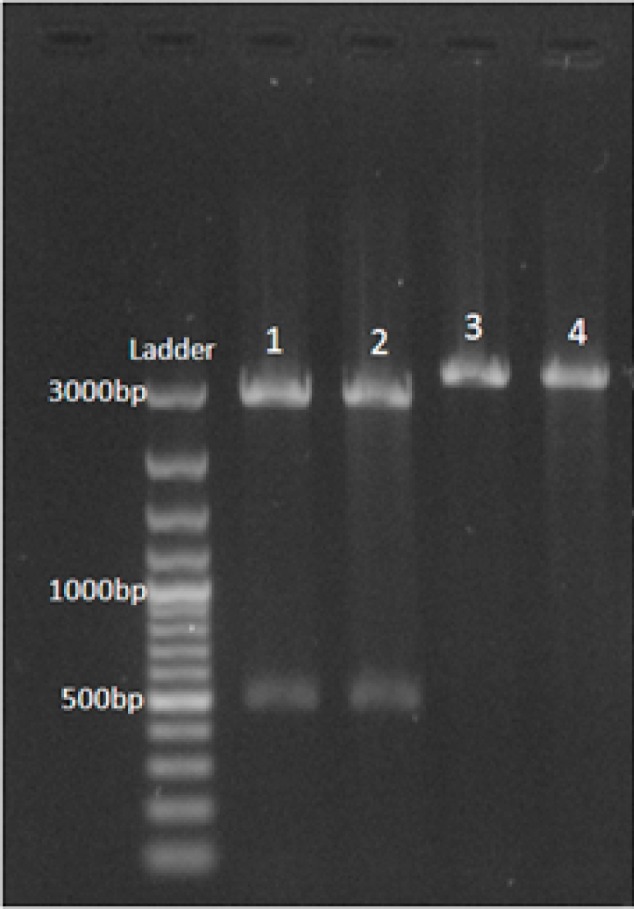
Digestion of pTZ57R/T plasmid containing EG95 fragment by *EcoRI* and *XhoI* restriction enzymes. Lane 1 & 2: double digestion, Lane 3 & 4: mono digestion

Total DNA and RNA were extracted from protoscoleces derived from sheep hydatid cysts and assessed by electrophoresis on the agarose gel. The extracted DNA and RNA (cDNA) were used as templates in PCR and RT-PCR reactions. The result is shown in Fig. 1. The RT-PCR product was successfully ligated into the pTZ57R/T plasmid and transformed to Top10 of *E.coli*. To confirm the ligation, the pTZ57R/T plasmid was digested with *EcoRI* and *XhoI* restriction enzymes (Fig. 2).

**Fig. 3:**
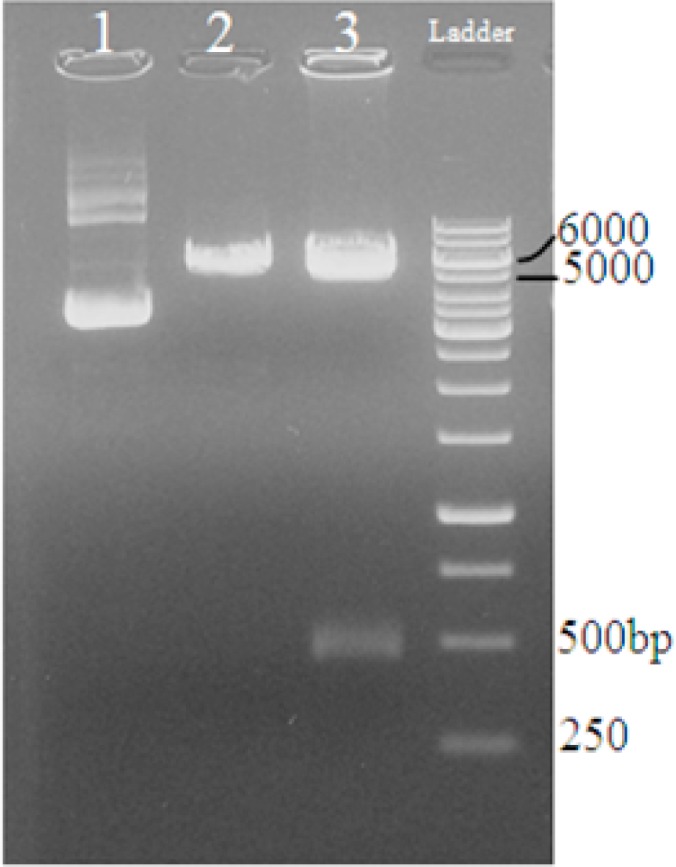
Digestion of pcEG95-IR plasmid by *EcoRI* and *XhoI* restriction enzymes. Lane 1: no digestion, Lane 2: mono digestion, Lane 3: double digestion

Then, the digested pT-Eg95 was ligated successfully into pcDNA3 plasmid (as expression vector) and transformed into Top10 strain of *E. coli*. The pcEG95 plasmid was digested by restriction enzymes to confirm ligation of this gene into pcDNA3 plasmid (Fig. 3).

In next step, pcEG95-IR plasmid and amplified DNA of EG95 were sequenced, and the sequences were submitted in GenBank with two-accession numbers (**JF357600.1**, **JF829212**). These sequences were compared with EG95 gene of *E. granulosus* in GenBank. The result showed 100% homology with *E. granulosus* EG95-5 and EG95-6 genes, which recorded in GenBank with the **AF199350.1** and **AF199347.1** accession numbers. In addition, DNA sequence was identical 99% with some sequences recorded in GenBank like **AF199349.1**, **EU595909.1** and **EU595882.1**.

**Fig. 4:**
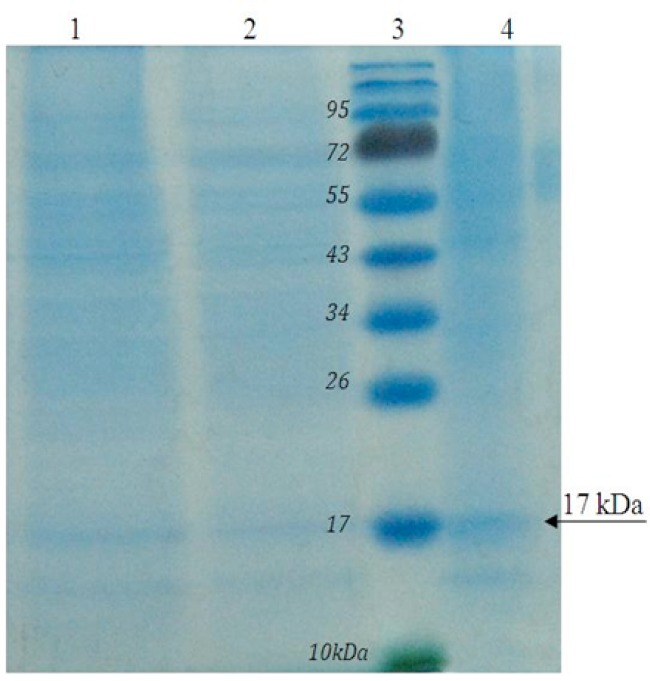
Electrophoresis of recombinant EG95 protein on SDS PAGE. Lane1: CHO cells without plasmid, Lane2: CHO cells with pcDNA3 plasmid, Lane3: Protein Ladder, Lane4: CHO cells with pcEG95-IR plasmid

**Fig. 5:**
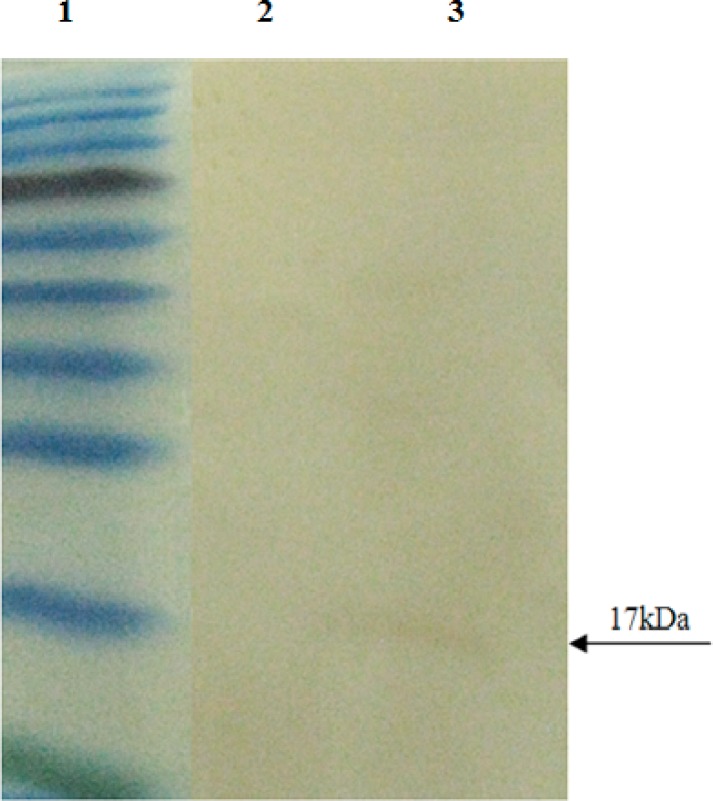
Western blot analysis of recombinant EG95 protein. Lane 1: protein molecular weight marker, Lane 2: CHO cells (negative control), Lanes 3, transfected cells containing pcDNA3 plasmid (17 kDa)

The expressed recombinant EG95 protein was evaluated by SDS PAGE and western blot, showing about 17kDa band on acrilamid gel (Fig. 4) and nitrocellulose membrane (Fig. 5).

## Discussion

Echinococcosis is a zoonotic disease with worldwide distribution caused by adult or larval stages of *Echinococcus* (family Taeniidae) (13). Controlling human hydatid disease using anthelmintics, simultaneously with changes in human life and animal managing have been unsuccessful in some developing countries (9). So, use of effective vaccine has been considered as the most efficient way to control echinococcosis.

Early attempt for vaccination the sheep against *E.granulosus* had been performed by Gemmell (1966), where he used oncosphere of the parasite as a crude antigen (14). Heath et al. (1981) and Osborn & Heath (1981) also showed that oncospheres have potent to prevent hydatid disease (15, 16). Later, Heath and Lawrence (1996) defined the antigenic polypeptides in oncosphere that induce protective immunity. Their results indicated that, only the fraction containing the 23 and 25 kDa molecules (EG95 protein) was able to stimulate protection against the infection (17).

EG95 is one of most important protein in *E.granulosus* and may be a vital function in parasite biology, which expresses in all stages of life cycle of this worm (18). This protein has high degree conservation in different stage of *Echinococcus* development. It may be involved in penetration of parasite to the epithelial border of the intestinal villi (19).

The EG95 encoding gene is a member of a multiple gene family, which expresses in the oncosphere, mature worm, and protoscoleces. Four EG95-related genes are expressing an identical EG95 protein (EG95-1, EG95-2, EG95-3, and EG95-4) only in the oncosphere life-cycle stage, as well as EG95-5 and EG95-6 additionally express in oncosphere, adult worm and protoscoleces (20, 21).

In the present work, we used gene coding EG95 protein in protoscoleces. Our results indicated that cDNA of EG95 in Iranian isolates of *E. granulosus* has 492bp and it identical with the sequences reported earlier (20) and 85% identical with the sequences reported by Lightowlers et al. (10). The difference may be due to the stage of parasite development, where Lightowlers et al. (1999) had used oncosphere for amplification of EG95 genes but in our work, we used protoscoleces.

In our study, the size of the recombinant EG95 protein demonstrated was about 17 kDa, which is different from the size of the native protein (23–25 kDa) reported by Heath and Lawrence (17). This discrepancy in size may be due to post-translational glycosylation (20).

On the other side, a number of studies in mice have shown that DNA vaccination can induce antibody and cell-mediated responses to a variety of bacterial, viral, and parasitic antigens (21). DNA vaccines considered to have potential advantages because of easier construction, ability to induce long-lasting immune responses, high temerature stability and low production cost (19).

In the present study, EG95 fragment successfully subcloned in pcDNA3 as eukaryotic expression vector to produce protein for DNA vaccine. Additionally, recombinant plasmid of pcEG95 was constructed successfully to be used for recombinant vaccine. In sheep, vaccination with recombinant EG95 protein induces high levels of protection against *E. granulosus* (7). The mechanism of protection appears to be strongly correlated with the presence of antibodies and the complement-mediated lysis of the parasite oncosphere (22).
